# Modified high dorsal procedure for performing isolated anatomic total caudate lobectomy (with video)

**DOI:** 10.1186/s12957-016-0896-3

**Published:** 2016-04-29

**Authors:** Toshiya Ochiai, Hiromichi Ishii, Atsushi Toma, Takeshi Ishimoto, Yusuke Yamamoto, Ryo Morimura, Hisashi Ikoma, Eigo Otsuji

**Affiliations:** Division of Digestive Surgery, Department of Surgery, Kyoto Prefectural University of Medicine, Kyoto, Japan; Department of Surgery, North Medical Center, Kyoto Prefectural University of Medicine, 481 Otokoyama, Yosano-cho, Yosa-gun, Kyoto, 629-2261 Japan

**Keywords:** Anatomical resection, Caudate lobectomy, High dorsal resection

## Abstract

**Background:**

Isolated anatomic total caudate lobectomy is indicated in patients who have liver tumors limited to the caudate lobe. However, isolated caudate lobe resection is a challenging surgical procedure that required safe and reliable techniques. All portal and hepatic veins that connect this area originate from the first branch of the portal vein or vena cava; therefore, the operator must be cautious of the potential for massive bleeding.

**Methods:**

The important points regarding the safety of our procedure include creating an optimal surgical view and preparing for accidental bleeding before parenchymal dissection. Sufficient mobilization and removal of Spiegel’s lobe from the left to the right side of the vena cava allows the operator to perform parenchymal dissection under a right- or front-side view.

**Results:**

We have performed this technique in two patients with HCC and one patient with primary cystadenocarcinoma. The average operative time and amount of blood loss were 435 min and 1137 ml, respectively. No operative mortalities or postoperative complications were observed in any of the patients. Our three patients are currently doing well without any recurrence.

**Conclusion:**

Our modified high dorsal resection procedure can be used to safely remove the entire caudate lobe.

**Electronic supplementary material:**

The online version of this article (doi:10.1186/s12957-016-0896-3) contains supplementary material, which is available to authorized users.

## Background

**Fig. 1:**
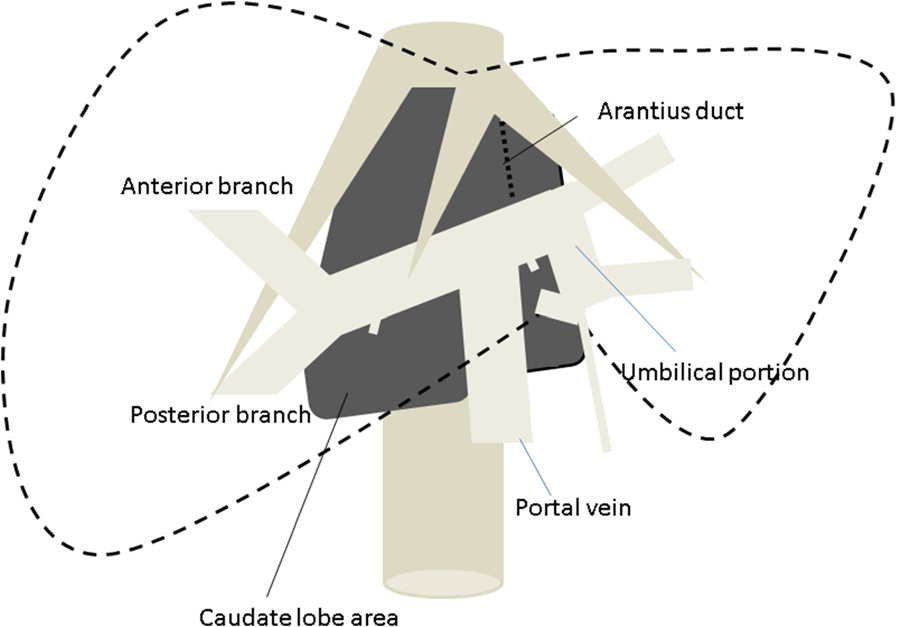
A scheme of the caudate lobe area

The caudate lobe is located in the deep dorsal area of the liver in front of the vena cava. This lobe consists of Spiegel’s lobe, the paracaval portion and the caudate process portion, which is bordered on the front side by the right and middle hepatic veins, on the back side by the vena cava and on the bottom side by a hilar plate (Fig. 1). Therefore, it is difficult to safely perform total caudate lobe resection due to accidental massive bleeding from the vena cava through short or long hepatic veins. Many surgeons have reported isolated caudate lobe resection to be a challenging surgical procedure that required safe and reliable techniques [1–3]. These anatomical and surgical obstacles are factors preventing surgeons from performing this procedure. Most patients who require total resection of the caudate lobe have previously received extended left or right lobectomy. However, for patients with liver dysfunction or a small tumor originating in the caudate lobe, performing extended lobectomy is sometimes impossible or considered to be excessive surgery. It is therefore necessary to perform isolated caudate lobectomy in such patients.

Anatomic total caudate lobe resection was first described by Takayama et al. as high dorsal resection of the liver in 1994 [4]. This procedure makes it potentially possible to remove the primary tumor in addition to histologically intrahepatic lesions spread throughout the portal branches of the caudate lobe. In this manuscript, we describe a safe technique for performing anatomic total caudate lobectomy of high dorsal resection approach.

## Methods

### Patient

#### Case 1

**Fig. 2:**
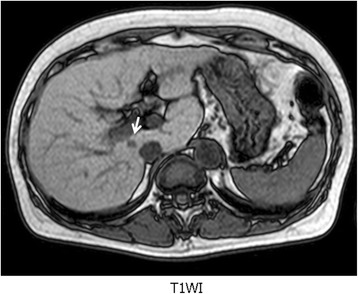
A small hepatocellular carcinoma (HCC) (diameter 5 mm) (*white arrow*) was detected in the paracaval caudate lobe of a 63-year-old female patient during a regular follow-up magnetic resonance imaging examination in 2012

A small hepatocellular carcinoma (HCC) (diameter 5 mm) was detected in the paracaval caudate lobe of a 63-year-old female patient during a regular follow-up magnetic resonance imaging examination in 2012 (Fig. 2). Her indocyanine green retention rate at 15 min was 6 %. She had sufficient liver function (Child-Pugh A) to undergo hepatectomy. In 2007, she had received combination therapy involving transarterial chemoembolization (TACE) and radiofrequency ablation for a solitary HCC in segment III and recovered. At first, she refused to undergo hepatectomy. So, TACE and percutaneous intrahepatic ethanol injection therapy were performed. However, the lesion was hypovascular and was too small to be punctured under ultrasonography for injection therapy. As the result, the non-surgical therapies were not effective. Therefore, we proposed a total caudate lobectomy, i.e., a high dorsal resection, in which the whole anatomical area including the small HCC would be removed. The high dorsal resection was performed using our safe procedure. The patient’s postoperative course was uneventful, and she was discharged on the 14th postoperative day. A pathological examination demonstrated that the nodule was a moderately differentiated HCC without ductal infiltration. The patient has not developed recurrence for 3 years.

#### Case 2 (cyst adenocarcinoma)

**Fig. 3:**
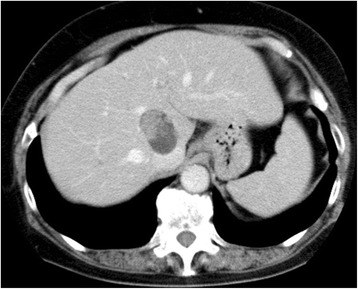
A cyst adenocarcinoma (diameter 4 cm) was accidentally detected in the caudate lobe of a 64-year-old female patient. The lesion consisted of a thick cystic capsule and papillary tumor without extra-capsule growth

A cyst adenocarcinoma (diameter 4 cm) was accidentally detected in the caudate lobe of a 64-year-old female patient by ultrasonography. The lesion consisted of a thick cystic capsule and papillary tumor without extra-capsule growth (Fig. 3). It was surrounded by hepatic veins and vena cava. Considering curability and remnant liver function, we proposed a high dorsal resection.

#### Case 3 (HCC)

**Fig. 4:**
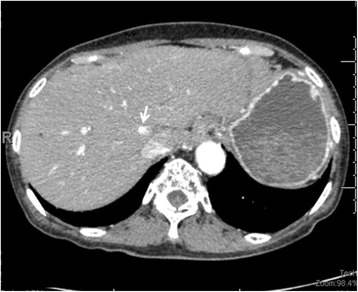
A small HCC (*white arrow*) was detected at the caudate lobe of an 80-year-old female patient with serum high alpha-feto-protein level, which was recognized only by arterial phase of dynamic computed tomography

A small HCC was detected at the caudate lobe of an 80-year-old female patient with serum high alpha-feto-protein level, which was recognized only by arterial phase of dynamic computed tomography (Fig. 4). Therefore, it was impossible to treat image-guided punctual therapies or seemed to be ineffective by TACE for this lesion. We proposed a high dorsal resection, in which the whole anatomical area including the small HCC would be removed.

### Surgical technique

Laparotomy was created via an inverted T-shaped incision in the upper abdomen. Before mobilizing the right and left lobes of the liver, we encircled the infrahepatic vena cava and secure it with rubber tape prepared for accidental bleeding. Then, Arantius’ ligament, the bilateral vena cava ligaments, and all short hepatic veins in the hepatic area of the vena cava were safely ligated and divided. This procedure created a free posterior surface of the caudate lobe. The roots of the right and middle-left hepatic veins were encircled by rubber tape prepared for bleeding from the branches of the hepatic veins at the site of hepatic parenchymal dissection. Following cholecystectomy, the right and left Glissonean pedicles at the hepatic hilar plates were also bluntly encircled with rubber tape (the Glissonean pedicle transection method) [5]. At this time, one or two portal branches extending to Spiegel’s lobe were ligated and separated. The cutting line of the border between the posterior section and paracaval portion or caudate process was determined according to the counterstain technique [6, 7].

**Fig. 5:**
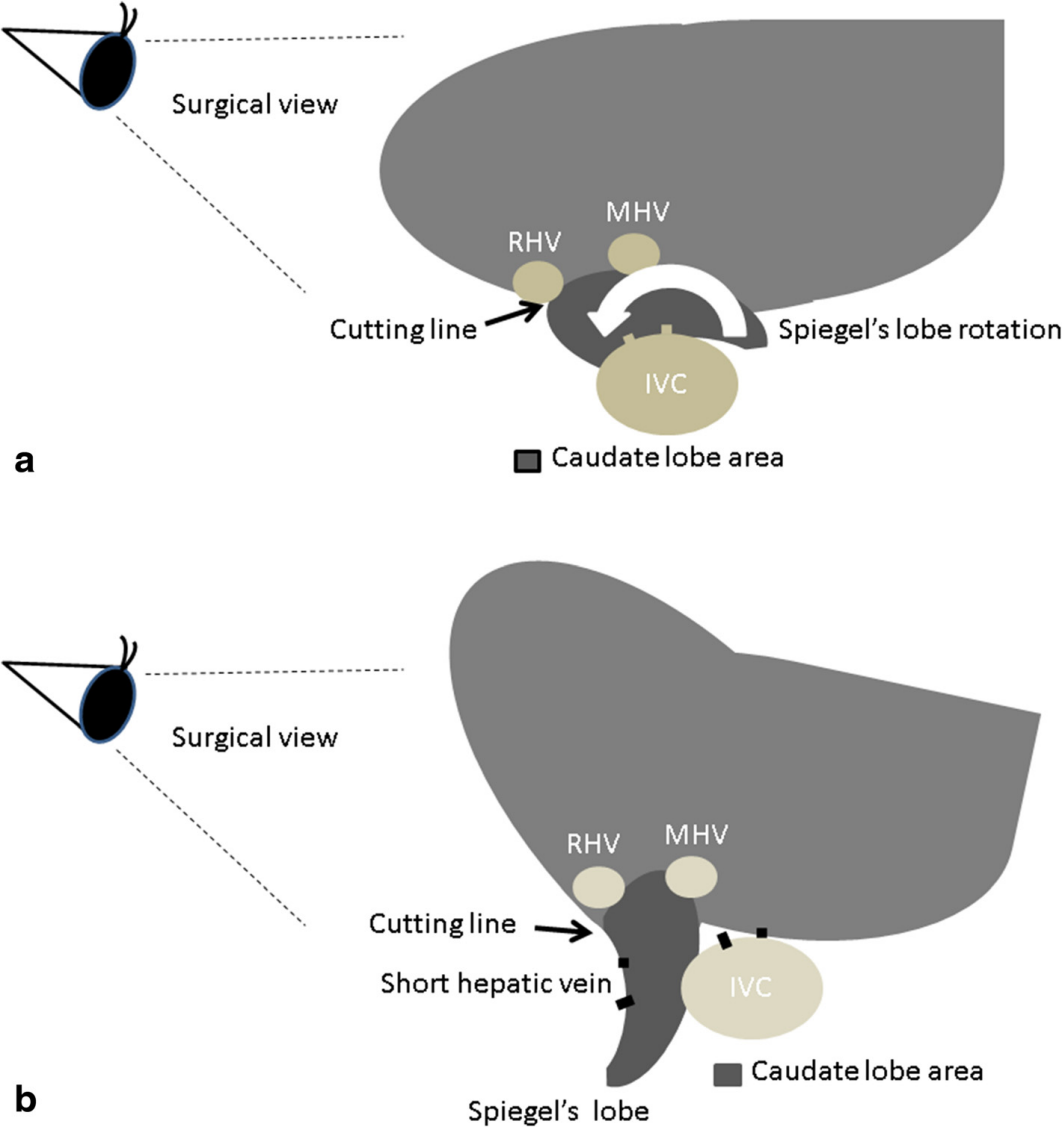
**a** It is difficult to visualize the cutting line for parenchymal dissection during high dorsal resection. **b** By rotating Spiegel’s lobe from the left to the right side of the vena cava, the cutting line for high dorsal resection can be easily visualized

Sufficient mobilization of the right and Spiegel’s lobe allowed the operator to easily dissect the parenchyma in this area due to the optimal surgical view. Pulling the tape encircling the Glissonean pedicles in the hepatic hilus, all Glisson’s capsules of the caudate lobe were ligated and divided. As a result, the bottom of the caudate lobe was freed from the hepatic hilum and the mobile area of the caudate lobe was enlarged. Spiegel’s lobe could be pulled out from the left side of the vena cava to the right side (Fig. 5a, b). Although the caudate lobe was normally located in a deep area of the abdomen, the cutting line was moved upward due to the technique, and the parenchyma could be dissected from the front or right side under an optimal surgical view. Parenchymal dissection was started beneath the right hepatic vein using an ultrasonic dissector according to Pringle’s method. The root of the middle hepatic vein was located near that of the right hepatic vein; therefore, the area beneath the middle hepatic vein could be easily exposed subsequent to that of the right hepatic vein. When the cutting line arrived at Arantius’ ligament, the entire caudate lobe was anatomically resected (Additional file 1).

### Ethic approval

This research was performed in accordance with the Declaration of Helsinki and was approved by the medical ethics committee of Kyoto Prefectural University of Medicine (RBMR-E-282-1). All of the patients agree that their clinical details and accompanying images are published.

## Results and discussion

We have performed this technique in two patients with HCC and one patient with primary cystadenocarcinoma and have had no experience with failing to remove Spiegel’s lobe, even in patients with histologic cirrhotic livers. The average operative time and amount of blood loss were 435 min and 1137 ml, respectively (Table 1). No operative mortalities or postoperative complications were observed in any of the patients. Although high dorsal resection requires a relatively long operative time, and a significant amount of intraoperative bleeding can occur compared with limited resection, the first priority is the postoperative prognosis of the patient. Our three patients are currently doing well without any recurrence.

**Table 1:** Clinical data of patients received nmodified high dorsal resection

Gender	Age	Disease tumor size	Operation	Op. time (min)	Op. bleeding (g)	Complication	Prognosis
Female (case l)	63	HCC 5 mm	High dorsal resection	284	730	–	36 months
							Alive
							No recurrence
Female (case 2)	64	Cyst adenocarcinoma 3 cm	High dorsal resection	580	1061	–	75 months
							Alive
							No recurrence
Female (case 3)	80	HCC 1 cm	High dorsal resection	441	1620	–	36 months
							Alive
							No recurrence

High dorsal resection involves theoretically sophisticated anatomical resection of the caudate lobe. This procedure is indicated in patients who have liver tumors limited to the caudate lobe. HCC of the caudate lobe is difficult to treat with image-guided punctual therapy or TACE. Additionally, according to recommendation of HCC treatment guidelines [8, 9], solitary HCC lesion should be removed by surgery. We have previously published a technique of mesohepatectomy with total caudate lobectomy [10]. This technique should be applied to basically huge tumors located at anterior or medial sections infiltrating to caudate lobe. Our modified high dorsal procedure is feasible for relatively small liver tumors without ductal infiltration, which limited to the caudate lobe. With regard to performing isolated anatomic total caudate lobectomy, anatomic resection has been superior to non-anatomic resection in postoperative prognosis of HCC [11, 12]. Theoretically, anatomic hepatectomy is the best way to prevent intrahepatic metastasis occurring via vascular invasion. However, the number of high dorsal resections has not significantly increased since 1994 [4]. Most liver tumors located in the caudate lobe are resected using limited resection or extended lobectomy. The rarity of cases indicated for this operation is one reason for the small number of procedures performed. Additionally, all portal and hepatic veins that connect this area originate from the first branch of the portal vein or vena cava; therefore, the operator must be cautious of the potential for massive bleeding. It is difficult to stop bleeding in this area because the row cut surface generally faces the area beneath the liver. This is another primary reason for the low number of operations. In order to overcome these obstacles, anterior hepatic transection for use during caudate lobectomy has been developed [13]. This procedure has been assessed to be safe, with an optimal surgical view. For huge liver tumors but limited to the caudate lobe, this anterior approach seems to be better than our modified high dorsal procedure [14]. However, the weakest point of this operation is the splitting of the liver parenchyma through Rex-Cantlie’s plane. Splitting takes time and results in bleeding during surgery. Several branches of the middle hepatic vein, which drain blood from the anterior or medial segments, are dissected during splitting. Moreover, splitting is performed after removing the caudate lobe.

**Fig. 6:**
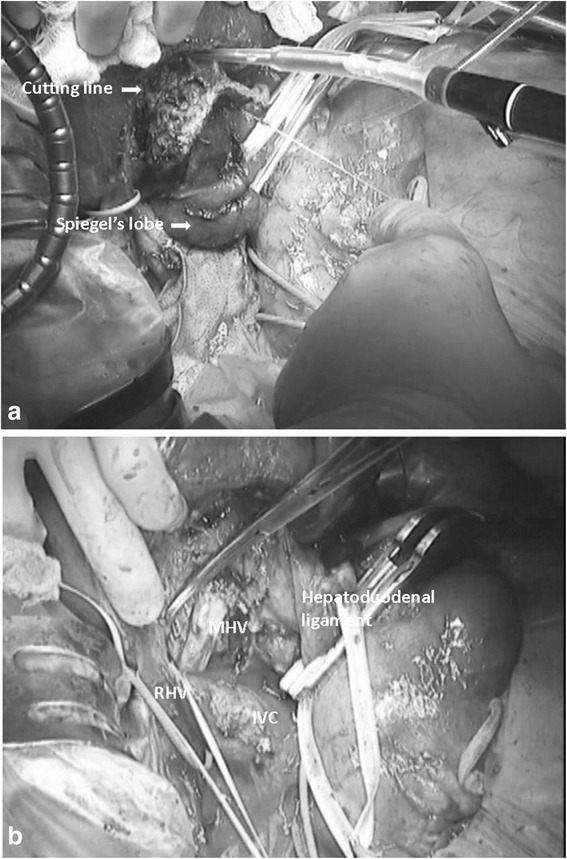
**a** The real picture of Spiegel’s lobe rotation. **b** The real picture of post caudate lobectomy. We can see the right and middle hepatic veins at the cutting surface

The important points regarding the safety of our procedure include creating an optimal surgical view and preparing for accidental bleeding before parenchymal dissection. Sufficient mobilization and removal of Spiegel’s lobe from the left to right side of the vena cava allows the operator to perform parenchymal dissection under a right- or front-side view. Therefore, we can dissect the right hepatic vein correctly and easily, which is the anatomic border of paracaval portion and segment VII. Moreover, we can also expose the middle hepatic vein, which is the anatomic border of caudate lobe and segment IV (Fig. 6a, b).

Laparoscopic hepatectomy may be suitable for this procedure to secure an optimal surgical view at parenchymal dissection of deep area. There have been some reports of laparoscopic caudate hepatectomy, but they are almost limited resection or laparoscopic combined caudate and left hemihepatectomy [15, 16]. For a left-sided position, techniques and devices to stop bleeding from the beneath of hepatic vein and vena cava and sufficient mobilization with laparoscopic devices are needed. Authors referred that this approach is safe and feasible only in selected patients and by hepatobiliary surgeon with abundant experience in laparoscopic liver surgery. There seems to be several steps to perform laparoscopic high dorsal resection safely as general surgery.

## Conclusions

Our modified high dorsal resection procedure can be used to safely remove the isolated entire caudate lobe.

## Consent

Written informed consent was obtained from all patients for publication of clinical details and accompanying images. A copy of the written consent is available for review by the Editor of this journal.

## Additional file

Additional file 1: Parenchymal dissection under a right- or front-side view : The cutting line was movedupward due to the technique, and the parenchyma could be dissectedfrom the right or front side under an optical surgical view. Parenchymal dissection started beneath the right hepatic vein using an ultrasonic dissector according to Pringle's method. The root of the middle hepatic vein was located near the right hepatic vein, therefore, the area beneath the middle hepatic vein could be easily exposed subsequent to that of the right hepatic vein. (MPG 10236 kb)

## Abbreviations

HCC: hepatocellular carcinoma
TACE: transarterial chemoembolization
